# Nobiletin Inhibits Non-Small-Cell Lung Cancer by Inactivating WNT/*β*-Catenin Signaling through Downregulating miR-15-5p

**DOI:** 10.1155/2021/7782963

**Published:** 2021-12-30

**Authors:** Sang Hyup Han, Jeong Hee Han, Wan Joo Chun, Sang Soo Lee, Hae Sung Kim, Jin Won Lee

**Affiliations:** ^1^Department of Surgery, Chuncheon Sacred Heart Hospital, College of Medicine, Hallym University, Chuncheon 24253, Gangwon-Do, Republic of Korea; ^2^Department of Pharmacology, College of Medicine, The Kangwon University of Korea, Chuncheon, Republic of Korea; ^3^Institute for Skeletal Aging & Orthopedic Surgery, Hallym University-Chuncheon Sacred Heart Hospital, Chuncheon, Republic of Korea

## Abstract

**Background:**

Nobiletin is a natural compound with anticancer activity; however, the mechanism is not clear.

**Methods:**

The inhibitory effect of nobiletin on non-small-cell lung cancer (NSCLC) cells was examined using soft agar, Transwell, and apoptosis analyses. Cancer stemness was measured by sphere assay. Genes and miRNAs regulated by nobiletin were identified by whole-genome sequencing. Protein levels were detected by western blot and immunofluorescence assays.

**Results:**

Nobiletin significantly inhibited NSCLC cell colony formation and sphere formation and induced apoptosis. Nobiletin upregulated negative regulators of WNT/*β*-catenin signaling, including NKD1, AXIN2, and WIF1, while it inhibited the expression of *β*-catenin and its downstream genes, including c-Myc, c-Jun, and cyclin D1. Furthermore, we identified that GN inhibits miR-15-5p expression in NSCLC cells and that NKD1, AXIN2, and WIF1 are the target genes of miR-15-5p.

**Conclusions:**

Nobiletin has a strong inhibitory effect on NSCLC, and nobiletin plays an anticancer role by inhibiting miR-15-5p/*β*-catenin signaling in NSCLC.

## 1. Introduction

Lung cancer is the most frequently diagnosed malignancy and the leading cause of cancer-related mortality worldwide [1–3]. Non-small-cell lung cancer (NSCLC) accounts for 80–85% of all lung cancers. Despite considerable advancements in diagnosis and treatment modalities, including surgery and chemoradiation therapy, the survival of patients with advanced stages of the disease remains unsatisfactory. Several oncogenes and tumor suppressor genes known to be associated with NSCLC have been investigated to analyze tumor behavior and determine their prognostic significance [4–6]. In this regard, there is a need to discover new agents for the treatment of this deadly disease. Many kinds of natural products have been proven to possess anticancer properties [7–9]. These types of components can affect the overall process of carcinogenesis by several mechanisms. Among them, nobiletin has been known to have an anticancer effect, as it can inhibit the proliferation, invasion, or metastasis of tumor cells. Nobiletin is a flavonoid compound isolated from the peels of citrus fruits, such as *Citrus unshiu* (Satsuma mandarin) and *Citrus sinensis*. Studies show that nobiletin exhibits an anticancer role in various cancers, including skin, prostate, colon, and breast cancer [10–18]. However, the anticancer mechanism of nobiletin is still unclear.

WNT/*β*-catenin signaling is a well-studied oncogenic pathway that plays an important role in the development and progression of various cancers. For example, there have been some reports that WNT/*β*-catenin might enhance the motility of malignant cells and tumor invasion, such as in breast cancer, melanoma, and gastric cancer [19–23]. As a result, many investigators have focused on the potential effect of some substances, such as flavonoids, on cancer prevention and therapy by inhibiting the WNT/*β*-catenin signaling pathway [1–3]. In fact, most reports show that inhibition of WNT/*β*-catenin signaling is a useful strategy for cancer treatment.

In this study, we demonstrated that nobiletin significantly inhibits NSCLC growth, metastasis, and stemness and stimulates NSCLC cell apoptosis. In addition, we demonstrated that nobiletin plays its role by inactivating Wnt/*β*-catenin signaling by upregulating the expression levels of WIF1, AXIN2, and NKD1. We also identified that nobiletin inhibits the expression of WIF1, AXIN2, and NKD1 by inhibiting miR-15-5p in NSCLC cells.

## 2. Materials and Methods

### 2.1. Reagents

Bicinchoninic acid (BCA) assay reagent was purchased from Bio-Rad (California, USA), Lipofectamine was purchased from Invitrogen (California, USA), the ECL (enhanced chemiluminescence) kit was purchased from Amersham Pharmacia (Buckinghamshire, UK), and the Immobilon-p membrane was purchased from Millipore (Massachusetts, USA). RPMI (Roswell Park Memorial Institute) 1640 medium, fetal bovine serum (FBS), TryPLE-Express enzyme, and Hank's balanced salt solution were obtained from Gibco (New York, USA).

### 2.2. Cell Lines and Cultures

Cell lines H460 and A549 were obtained from ATCC (Manassas, VA, USA), and these cells were cultured in DMEM (HyClone, Shanghai, China) supplemented with 10% FBS (ScienCell Research Laboratories, California, USA) in a 5% CO_2_ humidified atmosphere at 37°C. The cells were then treated with 2.5 *µ*M nobiletin for 24 hours and then subjected to a soft agar assay. After 10 days, the cell cultures were treated with 10 *µ*L of CCK-8 solution for 2 hours and incubated at 37°C for the determination of cell viability. A microplate reader was used to obtain the optical density (OD) at 40 nm. This research was approved by the Institutional Review Board of Chuncheon Sacred Heart Hospital.

### 2.3. Cell Viability and Soft Agar Assay

For the cell viability assay, H460 and H549 cells were seeded in 96-well plates at a density of 5000/well. The next day, the cells were treated with nobiletin or PBS for 48 h, and cell viability was measured using a Cell Counting Kit-8 (MedChem Express, JN, USA). For the soft agar assay, cells were treated with the indicated concentration of nobiletin for 48 h, and then, cells were resuspended in 0.5 mL of 0.35% agar (Sigma) in the growth medium at a density of 5000 cells/well in 6-well plates. The agar-cell mixture was plated on the top of a solid layer of 0.8% agar in the growth medium. Colonies were counted 14 days later.

### 2.4. *In Vitro* Migration and Invasion Assay

Cell migration and invasion assays were performed using a Transwell chamber. Cells were treated with the indicated concentration of nobiletin for 48 h. Then, the cells were resuspended, and 2 × 10^4^ cells in the serum-free medium were plated into the top chamber with or without gel coating. The cell growth medium with 10% FBS was added under the chamber. After 24 h, cells on the upper sides of the inserts were removed using cotton, and cells on the surface of the bottom sides of the inserts were fixed with 4% paraformaldehyde, followed by staining with 0.5% crystal violet. The cells in five random microscopic fields were photographed using an inverted phase-contrast microscope and counted.

### 2.5. Sphere Formation Assay

Cells were treated with the indicated concentration of nobiletin for 48 h, and then, a sphere formation assay was carried out as described in previous studies [17–20, 24]. In brief, cells were plated in Petri dishes (Corning, Tewksbury, MA, USA) at a concentration of approximately 10^4^ cells per ml of the sphere-forming medium consisting of a 1 : 1 mixture of high-glucose DMEM and Ham's Nutrient Mixture F-12 (Euroclone, Siziano, Italy) supplemented with 20 ng/ml EGF and 20 ng/ml recombinant FGFb, 1X Insulin-Transferrin-Selenium (Life Technologies, Carlsbad, CA, USA), 4 mg/ml bovine serum albumin (BSA, Sigma-Aldrich, Saint Louis, Missouri, USA), 2 mM glutamine (Euroclone), 50 U/ml penicillin, and 0.05 mg/ml streptomycin (Euroclone). After 6–7 days of growth, spheres were counted.

### 2.6. Luciferase Reporter Assay

The luciferase reporter assay was carried out as described by Fend [19].

### 2.7. Western Blot and RT-PCR Analyses

Western blot and RT-PCR analyses were carried out as described in previous studies [17–24]. For western blot analysis, equal amounts of whole-cell extracts were separated using SDS-PAGE, and the separated proteins were transferred to PVDF membranes. Membranes were incubated with the indicated primary and secondary antibodies. Protein bands were detected by enhanced chemiluminescence (Amersham Biosciences). For RT-PCR and real-time qRT-PCR analyses, total RNA was isolated from freshly dissected intestines using TRIzol reagent (Life Technologies), and complementary DAN synthesis was performed using an Omniscript Kit according to the manufacturer's instructions (QIAGEN). PCR amplification of miR-15-5p and RNU6 was performed with a specific primer set obtained from Thermo Fisher Scientific. PCR amplification of the following genes was performed with the QuantiTect SYBR Green method. The sequences of the primer pairs were as follows: GAPDH forward, 5′-GGAGCGAGATCCCTCCAAAAT-3′ and reverse, 5′-GGCTGTTGTCATACTTCTCATGG-3`; WIF1 forward, 5′-GTGTGAAATCAGCAAATGCC-3′, and reverse, 5′-GTCTTCCATGCCAACCTTCT-3; AXIN2 forward, 5′-AATTCGCGGGAGGGGGC-3′, and reverse, 5′-CTTCGTCGTCTGCTTGGTCAC-3`; NKD1 forward, 5′-TCGCCGGGATAGAAAACTACA-3′, and reverse, 5′-CAGTTCTGACTTCTGGGCCAC-3`.

### 2.8. Statistical Analysis

All data are presented as the mean ± standard deviation (SD), and significant differences (*P* < 0.05) between groups were analyzed by Student's *t*-test using SAS statistical software version 6.12 (SAS Institute).

## 3. Results

### 3.1. Nobiletin Inhibited the Growth of NSCLC *In Vitro*

**Figure 1:**
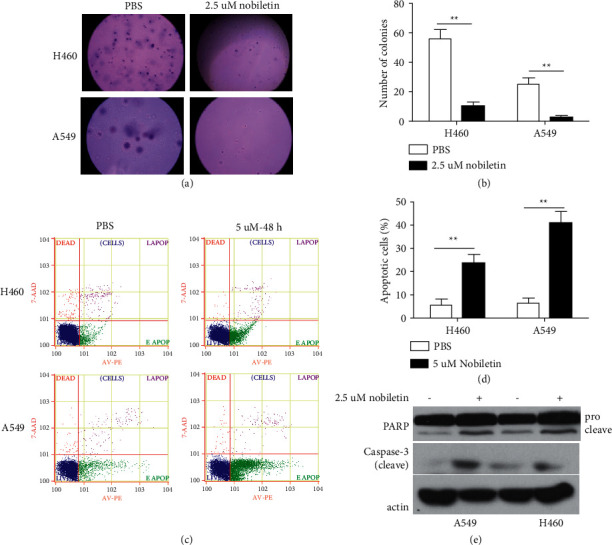
Nobiletin significantly inhibited NSCLC. (a, b) The inhibitory effects of nobiletin on NSCLC were measured by soft agar assay. (b, c) Apoptosis-promoting effects of nobiletin were measured by flow cytometry assay. (d) The effects of nobiletin on the expression of proapoptotic proteins were measured by western blot. ^*∗∗*^*P* < 0.01.

We first examined the inhibitory effects of nobiletin on NSCLC. Both H460 and A549 NSCLC cells were treated with nobiletin and then subjected to soft agar and apoptosis analyses. Our results showed that nobiletin significantly inhibited soft agar colony formation (Figures 1(a) and 1(b)) and significantly increased apoptosis in NSCLC cells (Figures 1(c) and 1(d)). Furthermore, western blot analysis confirmed the apoptosis-promoting effects of nobiletin on NSCLC. Western blot analysis showed that nobiletin significantly increased proapoptotic protein levels, including cleaved PARP and cleaved caspase 3 (Figure 1(e)). Taken together, our findings suggested that nobiletin inhibits NSCLC by stimulating apoptosis.

### 3.2. Nobiletin Inhibited the Invasion and Migration of NSCLC

**Figure 2:**
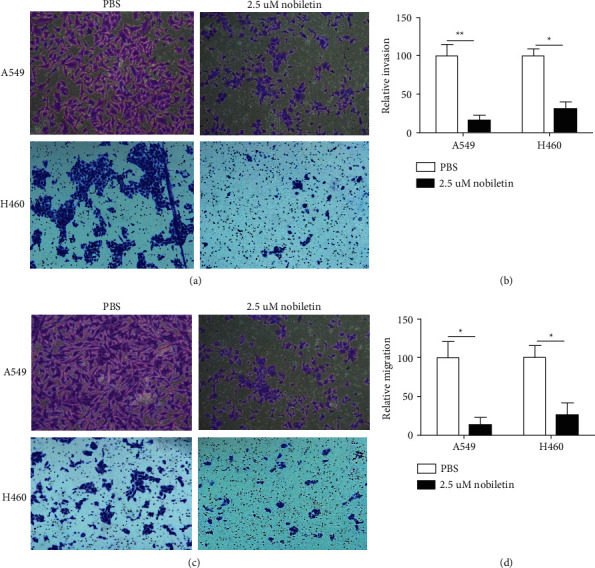
Nobiletin significantly inhibited the metastasis of NSCLC cells. (a, b) The effect of nobiletin on NSCLC cell invasion was measured. (c, d) The effect of nobiletin on NSCLC cell migration was measured.  ^*∗*^*P* < 0.05;  ^*∗*^ ^*∗*^*P* < 0.01.

Metastasis is a poor prognostic factor of NSCLC. Therefore, we investigated the effect of nobiletin on the invasion and migration of A549 and H460 cells. Transwell chamber assays were conducted to evaluate the effects of nobiletin on invasion and migration. As shown in Figures 2(a) and 2(b), nobiletin significantly suppressed the invasive capacities of both A549 (*P* < 0.01) and H460 (*P* < 0.05) NSCLC cells compared to their control group. Consistently, migration assays also showed that nobiletin significantly inhibited the migration abilities of both A549 and H460 cells (Figures 2(c) and 2(d)), suggesting that nobiletin has powerful antimetastatic effects.

### 3.3. Nobiletin Inhibits WNT/*β*-Catenin Signaling in NSCLC

**Figure 3:**
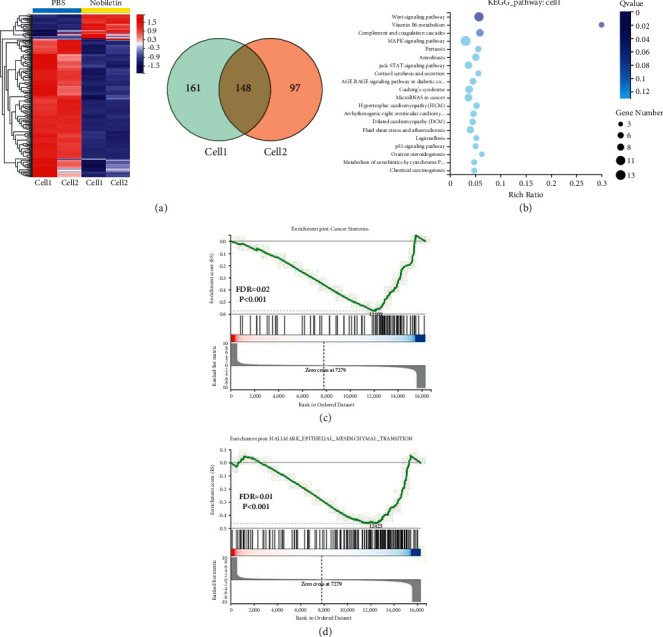
Nobiletin significantly affected WNT/*β*-catenin signaling in NSCLC. (a) Heatmap showing that nobiletin treatment caused alterations in the expression levels of many genes in A594 and H460 cells. (b) Kyoto Encyclopedia of Gene and Genomes (KEGG) analysis revealed that nobiletin treatment significantly affected cancer stemness maintenance-related WNT/*β*-catenin signaling. (c). GSEA showed that nobiletin treatment negatively correlated with epithelial-mesenchymal transition (EMT) and cancer stemness in NSCLC (d).

To investigate the anticancer molecular mechanism of nobiletin in NSCLC, A549 and H460 cells were treated with nobiletin for 48 hours and then subjected to whole-genome sequencing. Nobiletin treatment altered the expression levels of many genes in both NSCLC cell lines (Figure 3(a)). In addition, Kyoto Encyclopedia of Genes and Genomes (KEGG) analysis showed that nobiletin treatment significantly affected cancer stemness maintenance-related WNT/*β*-catenin signaling (Figure 3(b)). In fact, bioinformatics analysis of mRNA sequencing results showed that nobiletin upregulated negative regulators of WNT/*β*-catenin signaling, including NKD1, AXIN2, and WIF1 while inhibiting positive regulators of WNT/*β*-catenin signaling, including WNT6 and Jun (Table 1). Consistently, gene set enrichment analysis (GSEA) showed that nobiletin treatment was negatively correlated with epithelial-mesenchymal transition (EMT) and cancer stemness in NSCLC (Figure 3(c)). Together, these findings suggested that nobiletin has anticancer effects by inhibiting WNT/*β*-catenin signaling.

### 3.4. Nobiletin Inhibits Cancer Stemness and WNT/*β*-Catenin Signaling in NSCLC

**Figure 4:**
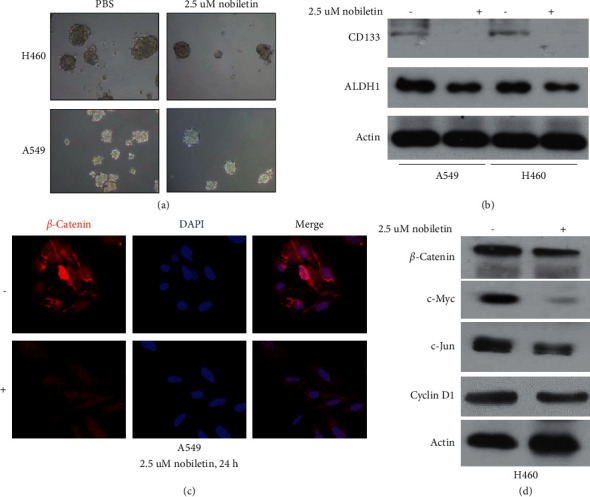
Nobiletin significantly inhibited cancer stemness and WNT/*β*-catenin signaling in NSCLC cells. (a) The effect of nobiletin on NSCLC cell stemness was investigated using a sphere formation assay. (b) The effect of nobiletin on NSCLC cell stemness was investigated by measuring cancer stemness marker protein expression by western blot. (c) The effect of nobiletin on WNT/*β*-catenin signaling was investigated by measuring *β*-catenin expression by IF. (d) The effect of nobiletin on WNT/*β*-catenin signaling was investigated by measuring the downstream gene expression of *β*-catenin signaling by western blot.

To investigate whether nobiletin is directly involved in the inhibition of cancer stemness and WNT/*β*-catenin signaling, NSCLC cells were treated with nobiletin and then subjected to sphere formation assays, and the expression levels of marker proteins of cancer stemness and WNT/*β*-catenin signaling were detected. Our results showed that nobiletin significantly inhibited sphere formation of NSCLC cells (Figure 4(a)) and cancer stemness-related marker protein expression (Figure 4(b)). In addition, nobiletin treatment significantly inhibited *β*-catenin levels (Figures 4(c) and 4(d)) and downstream protein expression levels of WNT/*β*-catenin signaling (Figure 4(d)). These results suggested that nobiletin is directly involved in the inhibition of cancer stemness and the WNT/*β*-catenin signaling pathway in NSCLC.

### 3.5. Nobiletin Suppressed WNT/*β*-Catenin Signaling by Downregulating miR-15-5p Expression in NSCLC

**Figure 5:**
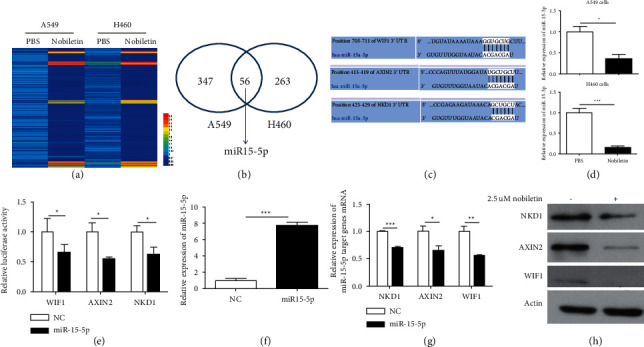
Nobiletin upregulates the negative regulator expression of WNT/*β*-catenin signaling and miR-15-5p expression. (a) Heatmap showing that nobiletin affected the expression levels of many miRNAs in NSCLC cells. (b) Nobiletin inhibited miR-15-5p expression in both A549 and H460 cells. (c) Predicted binding sites of miR-15-5p in the 3′-UTRs of NKD1, AXIN2, and WIF1. (d) The inhibitory effect of nobiletin on miR-15-5p expression was measured by qRT-PCR in NSCLC cells. (e) Luciferase activity of the reporter driven by the 3′-UTRs of NKD1, AXIN2, and WIF1 in A549 cells cotransfected with negative control oligonucleotides (NC) or miR-15-5p. (f) The transfection efficacy of miR-15-5p was measured by qRT-PCR. (g) The effects of miR-15-5p on the mRNA expression levels of NKD1, AXIN2, and WIF1 were measured by qRT-PCR. (h) The effects of miR-15-5p on the protein expression levels of NKD1, AXIN2, and WIF1 were measured by western blot.

To investigate the inhibitory mechanism of nobiletin on WNT/*β*-catenin signaling, we performed miRNA sequencing using nobiletin-treated NSCLC cells and their control cells because previous studies showed that miRNAs were involved in the regulation of WNT/*β*-catenin signaling by targeting their regulators [25]. miRNA sequencing data showed that the expression levels of many miRNAs were affected by nobiletin treatment (Figures 5(a) and 5(b)). Among them, we chose miR-15-5p for further experiments (Figure 5(b)) because miR-15-5p was inhibited by nobiletin, and miRNA database analysis showed that miR-15-5p can target negative regulators of WNT/*β*-catenin signaling, including NKD1, AXIN2, and WIF1 (Figure 5(c)). First, we confirmed the inhibitory effect of nobiletin on miR-15-5p expression by qRT-PCR. As expected, nobiletin treatment significantly inhibited miR-15-5p expression in NSCLC cell lines (Figure 5(d)). In addition, a luciferase reporter assay showed that overexpression of miR-15-5p inhibited the luciferase expression regulated by the 3′ UTRs of NKD1, AXIN2, and WIF1 (Figure 5(e)). Notably, qRT-PCR and western blot analysis showed that overexpression of miR-15-5p (Figure 5(f)) significantly inhibited NKD1, AXIN2, and WIF1 expression at both the mRNA (Figure 5(g)) and protein levels (Figure 5(h)).

**Figure 6:**
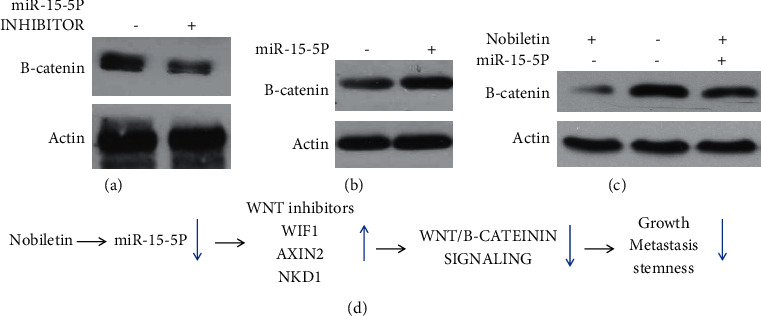
Nobiletin inhibits WNT/*β*-catenin signaling by inhibiting miR-15-5p. (a, b) The negative regulation of *β*-catenin expression by miR-15-5p was measured by western blot. (c) Western blot showing that miR-15-5p overexpression blocked nobiletin-induced inhibition of *β*-catenin expression in A549 cells. (d) A schematic model of the regulation of NSCLC progression by nobiletin.

Next, we investigated whether miR-15-5p was involved in nobiletin-induced inhibition of WNT/*β*-catenin signaling. As shown in Figures 6(a) and 6(b), inhibition of miR-15-5p inhibited the *β*-catenin expression level, while overexpression of miR-15-5p increased the *β*-catenin expression level. Notably, overexpression of miR-15-5p blocked nobiletin-induced inhibition of *β*-catenin expression (Figure 6(c)), suggesting that nobiletin inhibits WNT/*β*-catenin signaling through inhibition of miR-15-5p in NSCLC.

## 4. Discussion

In recent decades, natural products have received widespread attention as anticancer drugs. They have anticancer effects and fewer side effects [1–4]. However, natural products have not been widely used in clinical treatment because their mechanism of action is not clear. Nobiletin is a ubiquitous flavonoid derived from the peel of citrus fruits. Previous studies have demonstrated that nobiletin possesses therapeutic and biological activities, including antioxidant, anticancer, cardioprotective, and anti-inflammatory effects [10, 11, 13–18, 24], but the anticancer mechanism is still unclear.

Cancer stemness is a key factor for cancer progression. Studies show that increased cancer stemness stimulates cancer cell resistance to chemotherapy and enhances cancer cell invasiveness, thereby contributing to cancer progression [26], and increased cancer stemness also stimulates EMT [25]. EMT is a process in which epithelial tumor cells lose their cell polarity and cell-cell adhesion so that tumor cells gain migratory and invasive properties [27]. During EMT, epithelial cells lose epithelial markers, such as E-cadherin, which is the main molecule of stable epithelial adherens junctions, so that the invasion-metastasis cascade is promoted, resulting in the exit of tumor cells from the primary site, invasion to adjacent organs, intravasation, and distant metastasis via the blood or lymphatic system. Studies have shown that inhibition of cancer stemness and EMT can dramatically inhibit cancer progression [28, 29]. Abnormally increased cancer stemness and EMT are caused by many factors in cancer, and hyperactivation of WNT/*β*-catenin signaling is one of them [30, 31]. Here, our data showed that nobiletin inhibits NSCLC progression, cancer stemness, EMT, and WNT/*β*-catenin signaling, suggesting that nobiletin exerts its anticancer effects by inhibiting WNT/*β*-catenin signaling-regulated cancer stemness and EMT.

Here, we also demonstrated that WNT/*β*-catenin signaling was inactivated by nobiletin. WNT/*β*-catenin signaling is often hyperactivated in cancers by the downregulation of negative regulators, including NSCLC [32]. In addition, studies have shown that downregulated expression of the WNT/*β*-catenin signaling pathway is promoted by dysregulated miRNA expression in cancer [33]. Interestingly, studies have shown that natural products play an anticancer role by altering miRNA expression [34–36]. In this study, we reported for the first time that nobiletin suppresses miR-15-5p expression, thereby causing upregulation of miR-15-5p target genes, including negative regulators of WNT/*β*-catenin signaling, such as NKD1, AXIN2, and WIF1, which inactivate WNT/*β*-catenin signaling.

## 5. Conclusions

In conclusion, nobiletin inhibits NSCLC by inhibiting cancer stemness and EMT by inactivating the WNT/*β*-catenin signaling pathway in NSCLC. Nobiletin inhibited miR-15-5p expression, thereby increasing negative regulators of the WNT/*β*-catenin signaling pathway and ultimately inactivating the WNT/*β*-catenin signaling pathway. Our results shed light on the mechanism of the anticancer effect of nobiletin and provide more inspiration for physicians in treating patients with NSCLC.

## Figures and Tables

**Table 1:** Key genes related to WNT signaling that were affected by nobiletin treatment.

Gene	Expression	Role in WNT signaling
NKD1	Up	Inhibitor
AXIN2	Up	Inhibitor
WIF1	Up	Inhibitor
DKK1	Up	Inhibitor
WNT6	Down	Activator
JUN	Down	Activator, downstream gene

## Data Availability

All data generated or analyzed during this study are included within this article.
